# Pulse contour cardiac output monitoring is less reliable in critically ill children

**DOI:** 10.1186/cc10827

**Published:** 2012-03-20

**Authors:** JC Verheul, A Nusmeier, J Lemson

**Affiliations:** 1 Radboud University Nijmegen Medical Centre, Nijmegen, the Netherlands

## Introduction

Intermittent cardiac output measurement using the transpulmonary thermodilution (TPTD) method is considered to be the gold standard in young children but a validated continuous cardiac output technique is not available in these patients. We compared the continuous pulse contour cardiac output (PCCO) measurements with the TPTD method in critically ill children.

## Methods

We compared PCCO, measured with the PiCCO device (Pulsion, Munich, Germany), with TPTD measurements (CO_TPTD_) using the same device in a general pediatric intensive care (PICU) population. Because PCCO is calibrated with each TPTD measurement (CO_TPTD_) we compared the mean PCCO value just before a new TPTD measurement was done. We approved only CO_TPTD _measurement consisting of three consecutive TPTD measurements and we checked the thermodilution curve for a temperature difference of at least 0.2°C and a normal appearance. Only the intervals between two approved series of TPTD measurements were analysed. We calculated the correlation coefficient and used the Bland-Altman method for analysis.

## Results

**Figure 1:**
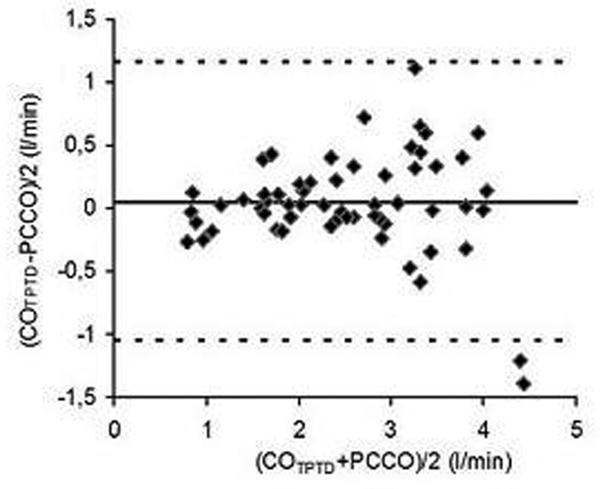
**Bland-Altman analysis of CO_TPTD _and PCCO**.

Sixty-one measurements in 10 children were included. Mean age was 24.5 (range 5 to 123) months; mean weight was 11.2 (range 3.8 to 18) kg, mean heart rate was 131/minute (range 87 to 193) and the mean blood pressure was 73 (range 49 to 96) mmHg. The mean CO_TPTD _was 2.60 (range 0.66 to 5.64) l/minute, mean cardiac index was 5.16 (range 2.76 to 10.83) l/minute/m^2 ^and mean duration of the interval was 5 hours and 33 minutes (range 14 minutes to 15 hours). The correlation coefficient between the CO_TPTD _and PCCO was 0.85 (*P *< 0.0001). The Bland-Altman analysis showed a mean bias of 0.06 l/minute (limits of agreement (LoA) ± 2.22 l/minute) (Figure 1). The percentage error was 43%. The correlation coefficient between the recalibration interval and the bias between CO_TPTD _and PCCO was -0.26 (*P *= 0.05). There was no correlation between CO_TPTD _and PCCO (*r *= 0.09 (*P *= 0.57)).

## Conclusion

The PCCO method cannot replace the transpulmonary thermodilution method in critically ill children.

